# ASAS Versus Modified New York Criteria for Ankylosing Spondylitis and Axial Spondyloarthritis: Attitudes and Utilization Among Saudi Rheumatologists

**DOI:** 10.3390/healthcare14203524

**Published:** 2026-10-20

**Authors:** Yousef M. Alammari, Abdullah Zaki Al-Fahd, Omar Ahmed Almukhalafi, Nasser Rashed Al-Tayar, Asim Mohammed Alshehri, Saud Abdullah Alsharif, Faisal Naif Almhesen, Rayan A. Qutob, Mohammad A. Alhajery, Abdulwahed A. Alotay, Abdulrahman Mohammed Alanazi, Yassir Daghistani

**Affiliations:** 1 Department of Internal Medicine, College of Medicine, Imam Mohammad Ibn Saud Islamic University (IMSIU), Riyadh 11432, Saudi Arabia; 2 College of Medicine, Imam Mohammad Ibn Saud Islamic University (IMSIU), Riyadh 11432, Saudi Arabia; 3 Department of Medicine, College of Medicine, University of Jeddah, Jeddah 23890, Saudi Arabia

**Keywords:** axial spondyloarthritis, ankylosing spondylitis, ASAS criteria, modified New York criteria, rheumatologists, classification criteria, Saudi Arabia

## Abstract

**Highlights:**

**What are the main findings?**

• Rheumatologists surveyed in Saudi Arabia rated the ASAS criteria as significantly more useful, and reported using them more frequently, than the modified New York criteria; 90.7% reported routinely ordering sacroiliac MRI when radiographs were negative.
• Inflammatory back pain and dactylitis were the features most frequently rated as “very specific”, and 51.9% of respondents perceived a need to modify the ASAS criteria.

**What are the implications of the main findings?**

• These self-reported patterns align directionally with a multinational survey of rheumatologists and may inform local teaching and referral pathways for axial spondyloarthritis.
• The high reported reliance on sacroiliac MRI highlights the need for studies of how the ASAS MRI definition is applied in local practice.

**Abstract:**

**Background/Objectives:** Axial spondyloarthritis (axSpA) is classified using two principal systems, the modified New York (mNY) and the Assessment of SpondyloArthritis international Society (ASAS) classification criteria. Although developed for research, both are widely used in clinical practice. We assessed the self-reported use of, and attitudes toward, both criteria sets among rheumatologists in Saudi Arabia, including the perceived need for their modification. **Methods:** A cross-sectional online survey was conducted using a 23-item questionnaire adapted from a published multinational survey. Wilcoxon signed-rank, chi-square goodness-of-fit, and Fisher’s exact tests were applied. **Results:** Among 54 respondents (estimated response rate, 22%), the ASAS criteria were rated significantly more useful (mean 4.26 vs. 3.85; Wilcoxon *p* = 0.001) and were used more frequently (Wilcoxon *p* < 0.001; high use, 75.9% vs. 57.4%) than the mNY criteria. When radiographs were negative, 90.7% reported ordering sacroiliac magnetic resonance imaging (MRI). Inflammatory back pain (77.8%) and dactylitis (63.0%) were most frequently rated as “very specific”. Overall, 79.6% considered the ASAS criteria sufficiently specific for clinical trials, while 51.9% perceived a need to modify them. No statistically significant associations were detected between demographic variables and the outcomes examined. **Conclusions:** Respondents reported greater use of, and higher perceived usefulness for, the ASAS than the mNY criteria, a pattern that aligns directionally with multinational survey data. These self-reported findings may inform local education and future studies of how classification criteria and sacroiliac MRI are applied in practice.

## 1. Introduction

Spondyloarthritis (SpA) is a cluster of chronic autoimmune inflammatory conditions marked by shared clinical and genetic features. These include involvement of the axial skeleton (sacroiliac joints and spine), asymmetric peripheral joint involvement predominating in the lower extremities, enthesitis, dactylitis, and extra-articular manifestations such as acute anterior uveitis, psoriasis, and inflammatory bowel disease, together with an association with the HLA-B27 antigen [1].

Axial spondyloarthritis (axSpA) encompasses a disease spectrum ranging from non-radiographic axSpA (nr-axSpA), in which structural changes are absent on plain radiographs, to radiographic axSpA (ankylosing spondylitis, AS) [2], defined by definitive sacroiliac joint damage on imaging [3]. Diagnosis can be challenging, as early disease may not produce detectable changes on conventional X-rays or magnetic resonance imaging (MRI). In individuals under 45 years presenting with chronic inflammatory back pain, a thorough evaluation including HLA-B27 testing, C-reactive protein measurement, and sacroiliac MRI is warranted [4].

Two classification systems are in widespread use. The modified New York (mNY) criteria require definite radiographic sacroiliitis alongside clinical features [5], so patients in the early, non-radiographic phase of disease cannot be classified with them; the delay from symptom onset to AS diagnosis has historically been reported to exceed seven years [6,7]. The Assessment of SpondyloArthritis international Society (ASAS) criteria, introduced in 2009, were developed to capture axSpA at an earlier stage through either an imaging arm (sacroiliitis on MRI or radiographs plus at least one SpA feature) or a clinical arm (HLA-B27 positivity plus at least two other SpA features), with a reported sensitivity and specificity of approximately 83% and 84%, respectively [8,9]. Both systems are classification criteria, developed to define homogeneous patient populations for research and clinical trials once a diagnosis has been established, rather than diagnostic criteria; in routine practice, however, they are frequently used to support diagnostic reasoning [10].

Despite their broader application, the ASAS criteria are not without limitations. The multi-component structure yields clinically heterogeneous patient groups, and false-positive MRI findings have been documented in athletes and postpartum women, raising concerns about overdiagnosis when the criteria are applied diagnostically [10,11,12,13]. A multinational survey by Rich-Garg et al. found that while two thirds of rheumatologists reported usually or always using the ASAS criteria to make a diagnosis, 42% felt they required modification [8], and in a recent survey of Moroccan community rheumatologists, 39.1% reported using the ASAS classification criteria as diagnostic criteria in their practice [14]. How classification criteria are used in practice therefore merits study in its own right. Saudi Arabia-specific data on this topic remain limited. This study aims to address that gap by describing the self-reported use of, and attitudes toward, both classification systems among rheumatologists in Saudi Arabia.

The Saudi context lends particular relevance to this question. HLA-B27, a cornerstone of the ASAS clinical arm, is less prevalent in Saudi Arabia than in populations of European descent: it was found in 2.3–2.5% of Saudi general-population samples and in 60.4% of Saudi axSpA patients, falling to 25.9% in those with nr-axSpA [15]. This comparatively low background prevalence may weaken the discriminatory value of the clinical arm and shift greater weight onto imaging. Compounding this, the management of axSpA in Saudi Arabia has been described as challenging owing to inadequate disease knowledge and, until recently, the unavailability of local guidelines [16]. Characterizing how Saudi rheumatologists use and perceive the two classification systems is therefore a useful first step toward their context-sensitive application.

## 2. Materials and Methods

### 2.1. Study Design, Setting, and Subjects

An online, survey-based cross-sectional study was conducted in the Kingdom of Saudi Arabia. Eligible participants were rheumatologists (consultants, specialists/registrars, and fellows) practicing in Saudi Arabia. The questionnaire was distributed through the official WhatsApp group of the Saudi Society for Rheumatology, which is routinely used to distribute research surveys to rheumatologists in Saudi Arabia; the group had approximately 250 members at the time of distribution and included fellows as well as consultants and specialists. For context, 246 rheumatologists were registered with the national rheumatology society in late 2021 [17], a figure similar to the size of the group, although this is an approximate comparison, as the group also included fellows and the national workforce has grown since 2021. Reminders were sent to the group on more than one occasion, and several potential respondents were also approached individually by members of the research team. One respondent declined consent and was excluded, yielding a final sample of 54 (estimated response rate, approximately 22%). No formal sample size calculation was performed, because the study aimed to survey all members of the professional network used for distribution rather than to test a pre-specified hypothesis.

### 2.2. Data Collection Instrument

A self-administered electronic questionnaire (Google Forms) was used (Supplementary Material S1). It was administered in English, the working clinical and academic language of rheumatology practice in Saudi Arabia, and was therefore not translated. The questionnaire was adapted from the 21-item survey developed by Rich-Garg et al. for a multinational study of rheumatologists in five countries; the original instrument was developed through several rounds of revision among its investigators and beta-tested for clarity of language among rheumatology division members at Oregon Health & Science University before use [8]. For the present study, the instrument was reviewed by two consultant rheumatologists practicing in Saudi Arabia, who assessed the relevance and wording of each item for the Saudi context. The core items on the usefulness and use of both sets of criteria, imaging practice, and the perceived specificity of SpA features were retained with essentially unchanged wording, and two items on the radiographic grading of sacroiliitis were not included. The other adaptations consisted of an opening consent item, locally relevant demographic items (nationality, professional grade, and region of practice in place of country), one additional item on whether respondents would treat nr-axSpA differently from radiographic axSpA, family history of SpA as an additional feature in the specificity ratings, and minor adjustments to wording and response options. The final instrument comprised 23 items: the consent item followed by six sections (general information and demographics, mNY criteria, ASAS criteria, radiological findings, features for diagnosing axSpA, and a final assessment that included opinions on criteria modification). Two items asked whether the current classification criteria (ASAS) require modification for axSpA (item 21) and for AS (item 22); because AS is classified by the mNY criteria, the wording of item 22 is imprecise, and the perceived need for modification is therefore reported from item 21, with item 22 shown for completeness (Section 3.4). No separate pilot test was conducted in the Saudi population.

### 2.3. Statistical Analysis

Continuous variables are presented as mean ± standard deviation (SD). Categorical variables are described using frequencies and percentages, with the denominator shown for each variable. Perceived usefulness was rated on a five-point Likert scale ranging from 1 (strongly disagree) to 5 (strongly agree), and frequency of use on a five-point scale ranging from 1 (never or rarely, <10%) to 5 (always, >90%); because both scales are ordinal, they are summarized as median (interquartile range, IQR) as well as mean ± SD. The Wilcoxon signed-rank test, a rank-based (non-parametric) test appropriate for ordinal data, was used to compare the perceived usefulness and frequency of use of ASAS versus mNY criteria within the same respondents (paired within-subject design), with effect sizes reported as *r* = *Z*/√*n*, where *n* is the number of paired responses (*n* = 54). Perceived specificity of each SpA feature was rated as very specific, somewhat specific, or non-specific, and features are ranked descriptively by the proportion of respondents rating each as “very specific”. The chi-square goodness-of-fit test was used only to assess whether the ratings for each feature departed from a uniform distribution across the three categories; it does not establish the diagnostic specificity of any feature. Fisher’s exact test was applied for all univariate comparisons between demographic subgroups and key outcome measures. Given the exploratory nature of these subgroup analyses, no formal correction for multiple comparisons was applied; as all comparisons were non-significant, such correction would not alter the conclusions. Multivariable logistic regression was not performed, as the sample size did not meet the minimum events-per-variable threshold (10 events per predictor) required for reliable model estimation. Missing data were handled by available-case analysis without imputation. The item on years since completing fellowship was addressed to consultants and specialists/registrars; it was answered by 49 of 54 participants, and the 5 who did not answer (1 consultant, 1 specialist/registrar, and 3 fellows) were excluded from analyses involving this variable. Four fellows answered this item despite its conditional wording; all selected “5 or less”, which is consistent with their training status, and their responses were retained. All other items, including the number of nr-axSpA diagnoses in the preceding 12 months, were answered by all 54 participants. A *p*-value < 0.05 was considered statistically significant. Statistical analyses were performed using IBM SPSS Statistics, version 27 (IBM Corp., Armonk, NY, USA).

### 2.4. Ethical Considerations

The study was conducted in accordance with the Declaration of Helsinki and approved by the Institutional Review Board of Imam Mohammad Ibn Saud Islamic University (approval number HAPO-01-R-0011, project no. 692/2024; date of approval 20 October 2024). All participants provided informed consent. Data confidentiality was maintained throughout, with access restricted to the study authors.

## 3. Results

### 3.1. Participant Characteristics

Of approximately 250 group members, 54 provided consent and completed the survey (estimated response rate, approximately 22%). Table 1 summarizes their sociodemographic and professional characteristics. The majority were male (61.1%), Saudi nationals (81.5%), and consultant rheumatologists (79.6%), and the mean age was 40.0 ± 6.63 years. The largest group practiced in the Central region (40.7%), and 31 of the 45 consultants and specialists who answered the item (68.9%) had completed fellowship within the past ten years. Women made up 38.9% of respondents, almost identical to the 39.0% reported among rheumatologists registered with the national society [17].

### 3.2. Perceived Usefulness and Utilization of Classification Criteria

**Figure 1:**
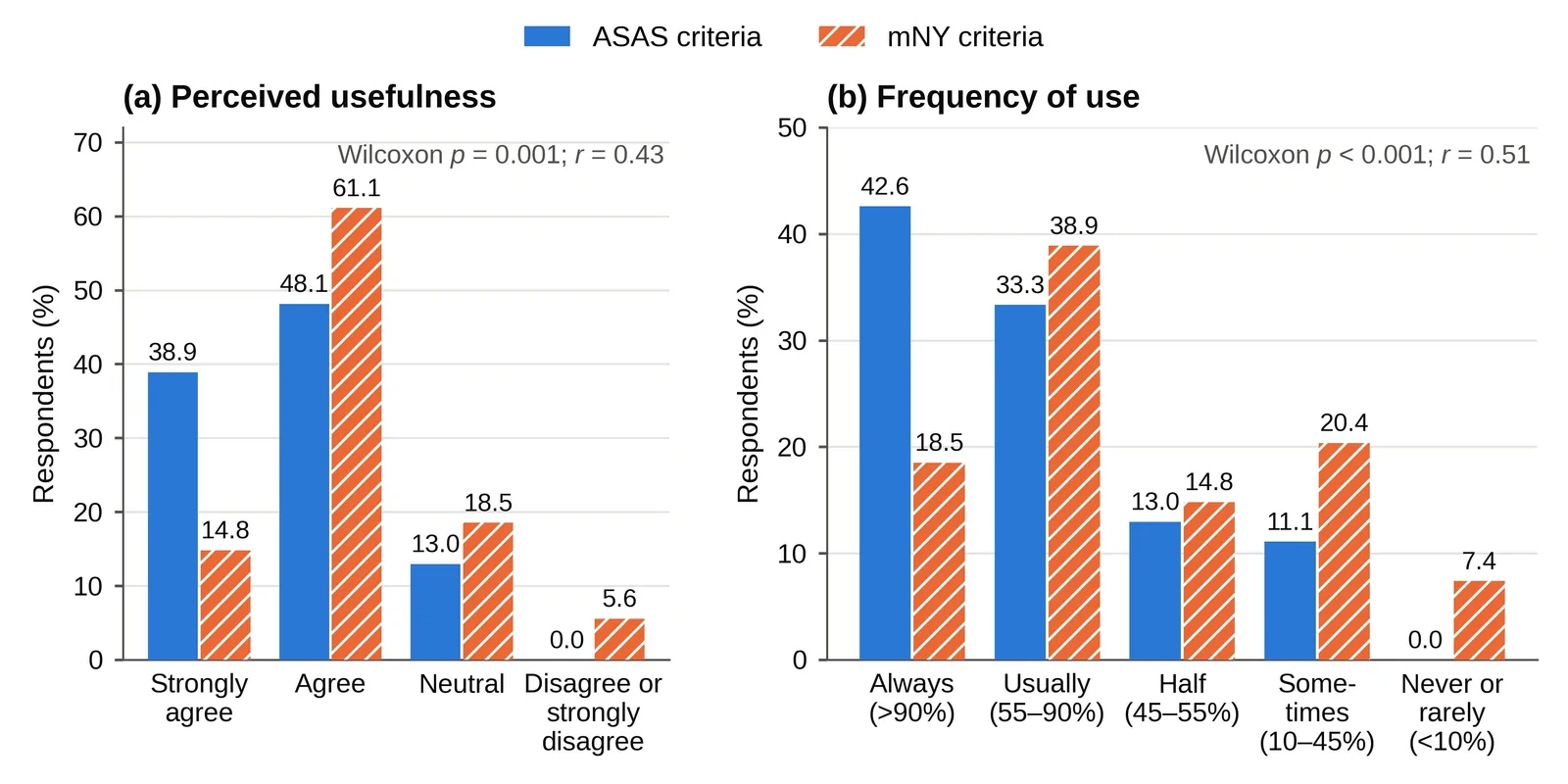
Self-reported perceived usefulness and frequency of use of the Assessment of SpondyloArthritis international Society (ASAS) and modified New York (mNY) classification criteria among 54 rheumatologists in Saudi Arabia (paired responses). (**a**) Perceived usefulness in clinical practice. (**b**) Frequency of use in clinical practice. Bars show the percentage of respondents in each category, with values shown above each bar. The ASAS criteria were rated as significantly more useful (*p* = 0.001; *r* = 0.43) and more frequently used (*p* < 0.001; *r* = 0.51) than the mNY criteria (Wilcoxon signed-rank test; *r*, effect size).

Table 2 and Figure 1 present a within-subject comparison of the ASAS and mNY criteria. The ASAS criteria were rated as significantly more useful than the mNY criteria (mean Likert score 4.26 ± 0.68 vs. 3.85 ± 0.74; median 4 [IQR 4–5] vs. 4 [IQR 4–4]; Wilcoxon signed-rank test, *p* = 0.001). Although the medians were identical, 20 respondents rated the ASAS criteria as more useful than the mNY criteria and 3 rated them as less useful (31 tied). No respondent rated the ASAS criteria as disagree or strongly disagree, compared with 5.6% for mNY. High use (always or usually) was reported by 75.9% for ASAS versus 57.4% for mNY (median 4 [IQR 4–5] vs. 4 [IQR 2–4]; *p* < 0.001); 24 respondents reported using the ASAS criteria more often than the mNY criteria and 3 less often (27 tied) (Figure 1).

### 3.3. Perceived Specificity of SpA Features

**Figure 2:**
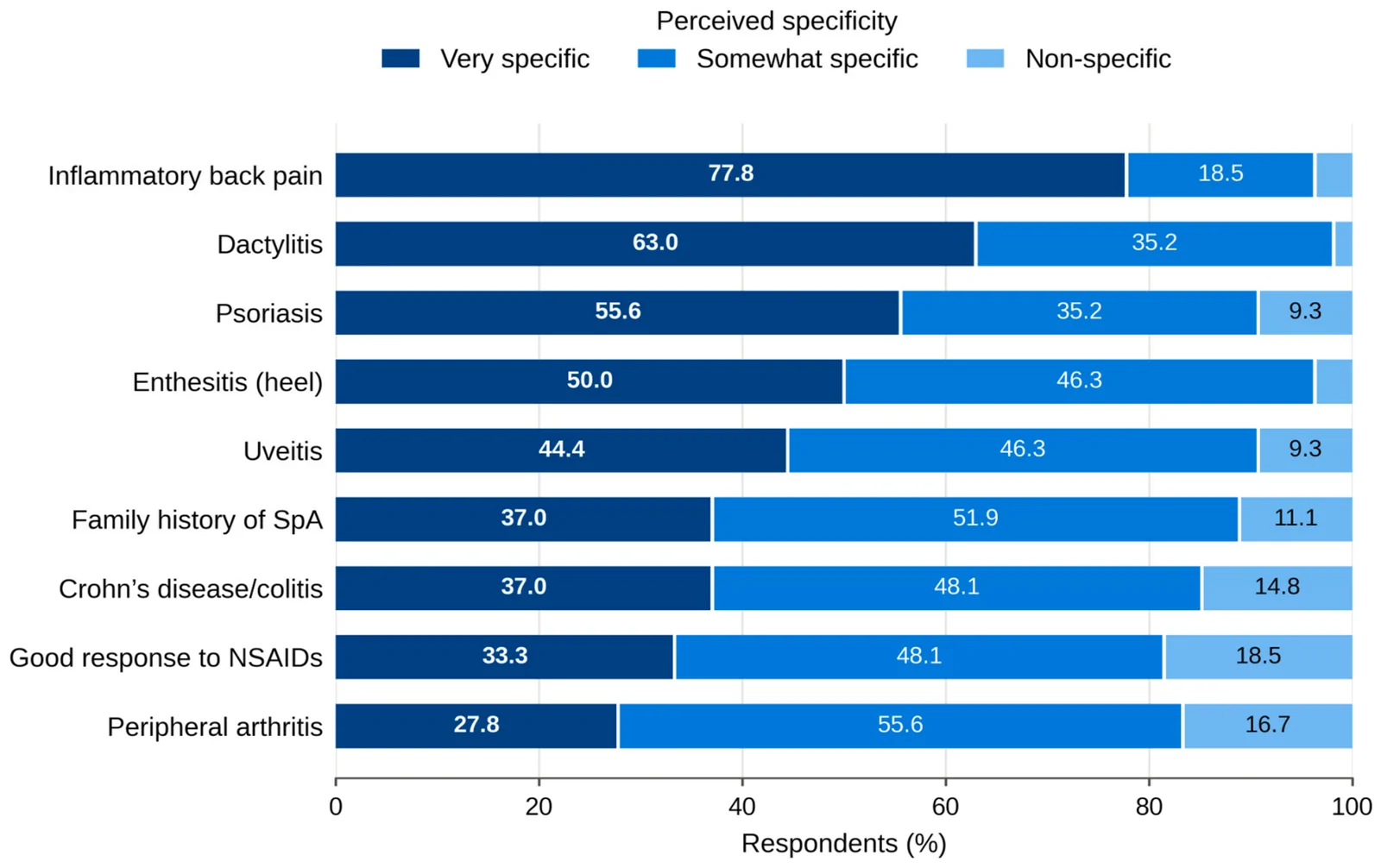
Perceived specificity of nine spondyloarthritis (SpA) features for axial spondyloarthritis (axSpA), as rated by 54 rheumatologists in Saudi Arabia. Features are ordered by the proportion of respondents rating each as “very specific”; values are percentages of respondents (values below 7% are not labeled; see Table 3). Inflammatory back pain and dactylitis were most frequently rated as “very specific”. Ratings reflect respondents’ perceptions rather than measured diagnostic specificity. NSAIDs, non-steroidal anti-inflammatory drugs.

Table 3 and Figure 2 present the perceived specificity ratings for nine SpA features, ranked by the proportion of respondents rating each feature as “very specific”. Inflammatory back pain was most frequently rated as very specific (77.8%), followed by dactylitis (63.0%) and psoriasis (55.6%). Good response to non-steroidal anti-inflammatory drugs (NSAIDs; 33.3%) and peripheral arthritis (27.8%) were least often rated as very specific; for both, “somewhat specific” was the most common rating. For all nine features, the distribution of ratings departed from a uniform distribution (chi-square goodness-of-fit test, all *p* < 0.05; Table 3).

### 3.4. Imaging Practices and Opinions on Criteria Modification

Table 4 summarizes self-reported imaging practices and opinions on the criteria; all items were answered by all 54 respondents. The majority (68.5%) reported assessing radiographs themselves with radiologist confirmation. MRI was the most frequently reported advanced imaging modality: 74.1% reported routinely ordering sacroiliac MRI even when sacroiliitis was visible on radiographs, and 90.7% when radiographs were negative, whereas computed tomography (CT) was rarely used. In the preceding 12 months, 31.5% reported having diagnosed five or more patients with nr-axSpA, 48.1% fewer than five, and 20.4% none. Overall, 79.6% agreed that the ASAS criteria are sufficiently specific for clinical trials, and 51.9% perceived a need to modify the ASAS criteria for axSpA (item 21); responses to the corresponding item on AS (item 22) were almost identical (51.9%), with 50 of 54 respondents giving the same answer to both items. The majority (70.4%) reported that they would not treat nr-axSpA differently from radiographic axSpA.

### 3.5. Univariate Analysis of Demographic Associations

Table 5 presents Fisher’s exact test results for comparisons between demographic variables (sex, clinical role, years since fellowship, and practice region) and key outcome measures. No statistically significant associations were detected (all *p* > 0.05). Given the overall sample size and the small size of several subgroups, these analyses were underpowered to detect modest differences; the absence of statistical significance does not establish equivalence between subgroups, and some numerical differences were observed (for example, high ASAS use was reported by 81.4% of consultants and 54.5% of other respondents; *p* = 0.109).

## 4. Discussion

In this cross-sectional survey of 54 rheumatologists in Saudi Arabia, respondents reported significantly greater use of the ASAS than the mNY classification criteria and rated them as more useful in clinical practice (*p* < 0.001 for frequency of use; *p* = 0.001 for perceived usefulness). To our knowledge, this is the first paired within-subject comparison of the two systems among rheumatologists in Saudi Arabia. The direction of this finding is consistent with the multinational survey by Rich-Garg et al., from which our instrument was adapted, in which 66% of respondents reported regular use of the ASAS criteria compared with 44% for the mNY criteria [8].

A plausible explanation for this pattern, consistent with previous studies, is that the mNY criteria require definite radiographic sacroiliitis and therefore do not capture patients in the early, non-radiographic phase of disease, whereas the ASAS criteria were designed to capture this earlier stage through an imaging arm or a clinical arm [9,18]. In the Spondyloarthritis Caught Early (SPACE) cohort of patients referred with chronic back pain, the mNY criteria showed a sensitivity of 16.9% and a specificity of 100% against the rheumatologist’s diagnosis, compared with 84.6% and 94.6%, respectively, for the ASAS axSpA criteria [19], and a meta-analysis reported a pooled sensitivity of 82% and a pooled specificity of 88% for the ASAS axSpA criteria [20]. The present survey did not assess the performance of either system.

A closer comparison with the multinational survey on closely matched items is informative (multinational survey vs. present study): regular use of the ASAS criteria, 66% vs. 75.9%; inflammatory back pain rated as very specific, 44% vs. 77.8%; dactylitis rated as very specific, 60% vs. 63.0%; sacroiliac MRI ordered when radiographs are normal, 83.6% vs. 90.7%; ASAS criteria considered sufficiently specific for clinical trials, 70% vs. 79.6%; and a perceived need to modify the current axSpA classification criteria, 42% vs. 51.9% [8]. The multinational figures are those reported in the published article and its supplementary material. In both studies, good response to NSAIDs and peripheral arthritis were the two features least often rated as very specific [8]. The proportions in our cohort were numerically higher on each of these items, most markedly for inflammatory back pain, although no formal between-study comparison was performed, and minor differences in item wording between the two instruments (for example, the source survey framed the imaging items as applying if reimbursement was not an issue) warrant caution in interpreting these differences.

Regional data remain limited. In a recent survey of 110 Moroccan community rheumatologists, 39.1% reported using the ASAS classification criteria as diagnostic criteria in their practice, and 91.8% reported managing nr-axSpA in the same way as radiographic disease [14], compared with 70.4% of our respondents who reported that they would not treat the two differently. The first of these findings underlines the importance of distinguishing classification from diagnosis when interpreting how the criteria are used in practice.

Despite the higher ratings given to the ASAS criteria, 51.9% of respondents perceived a need to modify them, a proportion somewhat higher than the 42% reported in the multinational survey [8]. The survey did not ask respondents why they held this view. Several authors have raised concerns that applying classification criteria diagnostically, particularly in settings with a low prevalence of axSpA, may contribute to overdiagnosis [10,11,21]; for example, sacroiliac bone marrow edema meeting the ASAS definition of a positive MRI has been reported in 30–41% of young athletes [12] and in 60% of women scanned within 10 days of delivery [13].

Reported reliance on MRI was high: 90.7% of respondents reported routinely ordering sacroiliac MRI when radiographs were negative. This is a descriptive finding; the survey did not examine MRI findings or their interpretation and cannot show whether this practice contributes to overdiagnosis. Concern about MRI-related misdiagnosis has, however, been reported by practicing rheumatologists elsewhere: in the Moroccan survey, 47.3% of respondents believed that sacroiliac MRI could lead to false-positive diagnoses [14]. A plausible explanation for the high reliance on imaging, not tested here, is the lower background prevalence of HLA-B27 in Saudi Arabia (2.3–2.5% in general-population samples), which may reduce the discriminatory value of the clinical arm of the ASAS criteria and shift weight toward imaging [15]. A similar dynamic has been described across North Africa and the Middle East, where variable access to and training in MRI, combined with low HLA-B27 frequency, complicates early and accurate diagnosis [22].

Inflammatory back pain was the feature most frequently rated as very specific (77.8%), followed by dactylitis (63.0%) and psoriasis (55.6%), whereas good response to NSAIDs and peripheral arthritis were least often rated as very specific. These ratings describe respondents’ perceptions and do not measure the diagnostic specificity of each feature. The relatively low ratings for NSAID response and peripheral arthritis mirror those in the multinational survey, whereas inflammatory back pain was rated as very specific more often in our cohort than in that survey (77.8% vs. 44%) [8].

Most respondents (70.4%) reported that they would not treat nr-axSpA differently from radiographic axSpA. This is consistent with the 2022 ASAS-EULAR (European Alliance of Associations for Rheumatology) management recommendations, which cover the full axSpA spectrum; in patients with high disease activity despite NSAIDs, they accept elevated C-reactive protein or sacroiliac MRI inflammation, and not only radiographic sacroiliitis, as a basis for starting biologic or targeted synthetic disease-modifying antirheumatic drugs [23]. In the Moroccan survey, recommendations for biologic therapy in nr-axSpA varied among respondents [14]. Our survey did not collect information on prescribing practice or on patient-level characteristics such as comorbidities; linking criteria use to treatment decisions, particularly biologic initiation in nr-axSpA, and examining comorbidity patterns through patient-level registry or chart-based studies are important directions for future work in Saudi Arabia.

These findings coincide with a wider regional movement toward locally adapted guidance: Saudi expert recommendations on early diagnosis and referral were published in 2022 [16], and a 2025 Arabian Gulf consensus subsequently produced region-tailored recommendations on referral, diagnosis, monitoring, and management [24]. Our findings provide quantitative evidence of the views reported by the participating rheumatologists, which may be relevant to such efforts.

Diagnostic delay remains an important concern: in a retrospective Saudi cohort from five centers, the median delay from symptom onset to axSpA diagnosis was 3 years (IQR, 1–6) [15]. Several referral strategies intended to identify patients with axSpA earlier have been evaluated, for example in the SPACE cohort [25]; whether the patterns of criteria use reported here influence diagnostic delay could not be assessed in this survey.

With the caveat that these findings describe self-reported attitudes and practice, three implications for practice emerge. First, the high reported use of the ASAS criteria indicates that they are already the system most widely used by the rheumatologists surveyed, which may inform local teaching, referral pathways, and future educational initiatives, including emphasis on their intended role as classification rather than diagnostic criteria. Second, the very high reported reliance on MRI when radiographs are negative raises the question of whether the ASAS MRI definition of active sacroiliitis is applied consistently in local practice; this was not assessed here and merits dedicated study, including the potential role of structured radiology reporting. Third, the proportion of respondents who perceived a need to modify the criteria indicates that international efforts to refine the classification criteria are relevant to local practice.

No statistically significant associations were detected between demographic variables and the outcomes examined (all *p* > 0.05). Because these analyses were underpowered to detect modest differences, particularly in small subgroups, this does not establish that views were equivalent across subgroups, and some numerical differences were observed (Table 5).

This study has several limitations. First, the sample was modest (*n* = 54), and no formal sample size calculation was performed, as the study aimed to survey all members of the professional network used for distribution. The sample size also precluded multivariable logistic regression, as the minimum events-per-variable threshold (10 EPV) was not met for any outcome, and the subgroup analyses were underpowered to detect modest differences: the absence of statistical significance does not establish equivalence between subgroups, and some numerical differences were present despite *p*-values above 0.05. Second, although the survey was distributed through the official WhatsApp group of the Saudi Society for Rheumatology, the response rate was approximately 22%. Physicians respond to surveys at lower rates than other groups (mean response rates of 54% for physicians and 68% for non-physicians in a review of mail surveys published in medical journals) [26], and response rates in comparable specialist surveys have also been low (approximately 6% in the multinational survey [8] and 25% in the Moroccan survey [14]), although the sampling frames differ and the rates are not directly comparable. Reasons for non-response were not collected; potential contributing factors include reliance mainly on a single voluntary online channel without incentives; the time needed to complete 23 items, most of which required clinical judgment; and competing clinical workload (lack of time was the main reason for non-response given by physician specialists in a web-based survey [27]). Selection and non-response bias therefore cannot be excluded: rheumatologists who are more engaged with classification criteria may have been more likely to respond, and because no information was available on non-respondents, formal non-response analysis was not possible, and the findings may not be generalizable to all rheumatologists practicing in Saudi Arabia. Although the group’s size was similar to the number of rheumatologists registered with the national society [17] and the proportion of women among respondents closely matched the national figure, no other respondent characteristic could be compared with published national data. Third, the adapted instrument underwent expert review by two consultant rheumatologists but was not pilot-tested locally or formally evaluated psychometrically in the Saudi population. Fourth, all data were self-reported and may diverge from actual practice, and social desirability bias cannot be excluded. In particular, the survey did not assess MRI positivity rates, the nature of MRI findings, reader expertise, the application of the ASAS MRI definition of active sacroiliitis, or concordance with the final clinical diagnosis; these questions require a dedicated imaging study. Finally, as a cross-sectional survey, the study captures attitudes at a single time point, in a limited number of practitioners, in one country; the findings are hypothesis-generating rather than confirmatory and cannot establish temporal trends or causal relationships. Future work should pursue larger samples recruited through multiple channels, together with formal piloting and validation of the instrument and correlation of stated attitudes with objective diagnostic and imaging-ordering data. Despite these limitations, responses were obtained from rheumatologists in several regions of Saudi Arabia and spanned consultants, specialists, and fellows, and the findings are directionally consistent with international survey data.

## 5. Conclusions

In this survey, rheumatologists in Saudi Arabia reported greater use of, and higher perceived usefulness for, the ASAS than the mNY classification criteria; frequent use of sacroiliac MRI when radiographs were negative; and perceived specificity ratings in which inflammatory back pain and dactylitis were most often rated as very specific. About half perceived a need to modify the ASAS criteria. These findings align directionally with a multinational survey of rheumatologists, with the same greater use of the ASAS than the mNY criteria, the same reliance on MRI when radiographs are normal, and the same two features least often rated as very specific (good response to NSAIDs and peripheral arthritis), while the proportions in our cohort were numerically higher for ASAS use and perceived need for modification. As self-reported, cross-sectional data from a single national sample, they are hypothesis-generating and support further study of how classification criteria and sacroiliac MRI are applied in practice.

## Figures and Tables

**Table 1:** Sociodemographic and professional characteristics of participating rheumatologists (*N* = 54).

	*n*	%
Sex		
Female	21	38.9
Male	33	61.1
Age (years)		
Mean ± SD	40.0 ± 6.63	
31–40 years	33	61.1
41–50 years	17	31.5
≥51 years	4	7.4
Nationality		
Saudi	44	81.5
Non-Saudi	10	18.5
Clinical Role		
Consultant rheumatologist	43	79.6
Specialist/Registrar	4	7.4
Fellow	7	13.0
Years Since Fellowship ^†^		
≤5 years	19	35.2
6–10 years	16	29.6
11–15 years	7	13.0
≥16 years	7	13.0
Not answered ^†^	5	9.3
Practice Region		
Central	22	40.7
Western & Southern ^‡^	18	33.3
Eastern	14	25.9
Primary Practice Focus		
Exclusively patient care	23	42.6
Patient care >75% (with research/teaching)	30	55.6
Research >75%	1	1.9

^†^ Item addressed to consultants and specialists/registrars; not answered by 1 consultant, 1 specialist/registrar, and 3 fellows. Four fellows who answered the item are included in the ≤5 years category. ^‡^ No respondents reported practicing in the Northern region; the Western (*n* = 16) and Southern (*n* = 2) regions are combined. SD, standard deviation.

**Table 2:** Comparison of perceived usefulness and frequency of use: ASAS versus modified New York (mNY) criteria (*N* = 54, paired responses).

	ASAS *n*	ASAS %	mNY *n*	mNY %
Perceived Usefulness in Clinical Practice				
Strongly agree	21	38.9	8	14.8
Agree	26	48.1	33	61.1
Neutral	7	13.0	10	18.5
Disagree/Strongly disagree	0	0.0	3	5.6
Mean score ± SD *	4.26 ± 0.68		3.85 ± 0.74	
Median (IQR) *	4 (4–5)		4 (4–4)	
*p *-value (Wilcoxon signed-rank) ^†^	*p *= 0.001			
Frequency of Use in Clinical Practice				
Always (>90%)	23	42.6	10	18.5
Usually (55–90%)	18	33.3	21	38.9
Half (45–55%)	7	13.0	8	14.8
Sometimes (10–45%)	6	11.1	11	20.4
Never/Rarely (<10%)	0	0.0	4	7.4
High use: Always + Usually	41 (75.9%)		31 (57.4%)	
Mean score ± SD ‡	4.07 ± 1.01		3.41 ± 1.22	
Median (IQR) ‡	4 (4–5)		4 (2–4)	
*p *-value (Wilcoxon signed-rank) ^†^	*p *< 0.001			

* Scored from 1 (strongly disagree) to 5 (strongly agree). ^†^ Wilcoxon signed-rank test for paired responses (*n* = 54). Although the medians are identical, 20 respondents rated the ASAS criteria as more useful than the mNY criteria and 3 as less useful (31 tied), and 24 reported using the ASAS criteria more often and 3 less often (27 tied). ^‡^ Scored from 1 (never or rarely, <10%) to 5 (always, >90%). ASAS, Assessment of SpondyloArthritis international Society; mNY, modified New York; SD, standard deviation; IQR, interquartile range.

**Table 3:** Perceived specificity of SpA features for axSpA as rated by respondents (*N* = 54), ranked by the proportion rating each feature as “very specific”.

	Non-Specific *n* (%)	Somewhat Specific *n* (%)	Very Specific *n* (%)	*N*	χ^2^	*p*-Value
Inflammatory back pain	2 (3.7)	10 (18.5)	42 (77.8)	54	49.78	<0.001
Dactylitis	1 (1.9)	19 (35.2)	34 (63.0)	54	30.33	<0.001
Psoriasis	5 (9.3)	19 (35.2)	30 (55.6)	54	17.44	<0.001
Enthesitis (heel)	2 (3.7)	25 (46.3)	27 (50.0)	54	21.44	<0.001
Uveitis	5 (9.3)	25 (46.3)	24 (44.4)	54	14.11	<0.001
Family history of SpA	6 (11.1)	28 (51.9)	20 (37.0)	54	13.78	0.001
Crohn’s disease/colitis	8 (14.8)	26 (48.1)	20 (37.0)	54	9.33	0.009
Good response to NSAIDs	10 (18.5)	26 (48.1)	18 (33.3)	54	7.11	0.029
Peripheral arthritis	9 (16.7)	30 (55.6)	15 (27.8)	54	13.00	0.002

Features are ranked by the proportion of respondents rating each as very specific (ties are ordered by the proportion rating the feature as non-specific, from lowest to highest). χ^2^, chi-square goodness-of-fit statistic (2 degrees of freedom) testing whether ratings departed from a uniform distribution across the three categories; it does not measure diagnostic specificity. SpA, spondyloarthritis; axSpA, axial spondyloarthritis; NSAIDs, non-steroidal anti-inflammatory drugs.

**Table 4:** Self-reported imaging practices and opinions on the ASAS classification criteria (*N* = 54).

	*n*	%
X-ray Sacroiliitis Assessment Approach		
Self-read + radiologist confirmation	37	68.5
Self-read only	9	16.7
Mainly radiologist report	8	14.8
MRI Ordered When Sacroiliitis IS Seen on X-ray		
Yes	40	74.1
No	14	25.9
MRI Ordered When Sacroiliitis NOT Seen on X-ray		
Yes	49	90.7
No	5	9.3
CT Scan Ordered (either scenario)		
Sacroiliitis seen on X-ray	1	1.9
Sacroiliitis NOT seen on X-ray	3	5.6
nr-axSpA Diagnoses in the Past 12 Months		
None	11	20.4
<5 patients	26	48.1
≥5 patients	17	31.5
ASAS Criteria Specific Enough for Clinical Trials		
Strongly agree + Agree	43	79.6
Neutral	9	16.7
Disagree	2	3.7
Perceived Need to Modify ASAS Criteria for axSpA (Item 21)		
Strongly agree + Agree	28	51.9
Neutral	22	40.7
Disagree	4	7.4
Perceived Need to Modify ASAS Criteria for AS (Item 22) *		
Strongly agree + Agree	28	51.9
Neutral	22	40.7
Disagree	4	7.4
Would Treat nr-axSpA Differently than Radiographic axSpA		
No	38	70.4
Yes	16	29.6

All items were answered by all 54 respondents. * Item 22 asked about the ASAS criteria in relation to ankylosing spondylitis (AS), which is classified by the mNY criteria; the wording is therefore imprecise, and the item is shown for completeness (50 of 54 respondents gave the same answer to items 21 and 22). No respondent selected “strongly disagree” for these items. ASAS, Assessment of SpondyloArthritis international Society; mNY, modified New York; axSpA, axial spondyloarthritis; nr-axSpA, non-radiographic axSpA; MRI, magnetic resonance imaging; CT, computed tomography.

**Table 5:** Univariate analysis of associations between demographic characteristics and key outcome measures (Fisher’s exact test; *N* = 54 unless otherwise indicated).

Outcome	Predictor	Group A *n*/*N* (%)	Group B *n*/*N* (%)	*p*-Value
High ASAS Use (Always or Usually)				
	Sex	Male: 25/33 (75.8%)	Female: 16/21 (76.2%)	1.000
	Clinical Role	Consultant: 35/43 (81.4%)	Non-Consultant: 6/11 (54.5%)	0.109
	Years since fellowship †	≤5 years: 16/19 (84.2%)	>5 years: 23/30 (76.7%)	0.720
	Region	Central: 14/22 (63.6%)	Other: 27/32 (84.4%)	0.109
High mNY Use (Always or Usually)				
	Sex	Male: 21/33 (63.6%)	Female: 10/21 (47.6%)	0.273
	Clinical Role	Consultant: 27/43 (62.8%)	Non-Consultant: 4/11 (36.4%)	0.173
	Years since fellowship ^†^	≤5 years: 13/19 (68.4%)	>5 years: 15/30 (50.0%)	0.247
	Region	Central: 12/22 (54.5%)	Other: 19/32 (59.4%)	0.784
Desire to Modify ASAS Criteria (Agree/Strongly agree)				
	Sex	Male: 18/33 (54.5%)	Female: 10/21 (47.6%)	0.781
	Clinical Role	Consultant: 24/43 (55.8%)	Non-Consultant: 4/11 (36.4%)	0.320
	Years since fellowship ^†^	≤5 years: 8/19 (42.1%)	>5 years: 20/30 (66.7%)	0.139
	Region	Central: 14/22 (63.6%)	Other: 14/32 (43.8%)	0.176
Would Treat nr-axSpA Differently (Yes)				
	Sex	Male: 10/33 (30.3%)	Female: 6/21 (28.6%)	1.000
	Clinical Role	Consultant: 13/43 (30.2%)	Non-Consultant: 3/11 (27.3%)	1.000
	Years since fellowship ^†^	≤5 years: 5/19 (26.3%)	>5 years: 10/30 (33.3%)	0.754
	Region	Central: 6/22 (27.3%)	Other: 10/32 (31.3%)	1.000
ASAS Criteria Specific Enough for Clinical Trials (Agree/Strongly agree)				
	Sex	Male: 27/33 (81.8%)	Female: 16/21 (76.2%)	0.733
	Clinical Role	Consultant: 33/43 (76.7%)	Non-Consultant: 10/11 (90.9%)	0.426
	Years since fellowship ^†^	≤5 years: 15/19 (78.9%)	>5 years: 23/30 (76.7%)	1.000
	Region	Central: 17/22 (77.3%)	Other: 26/32 (81.3%)	0.743

Values are *n*/*N* (%) within each subgroup. ^†^ Available for 49 respondents (item addressed to consultants and specialists/registrars; not answered by 5 respondents, including 3 fellows). Non-consultant: specialists/registrars and fellows; Other region: Western, Eastern, and Southern regions. No comparison reached statistical significance; the analysis was underpowered to detect modest differences, particularly in small subgroups, and non-significant results should not be interpreted as evidence of equivalence. ASAS, Assessment of SpondyloArthritis international Society; mNY, modified New York; nr-axSpA, non-radiographic axial spondyloarthritis.

## Data Availability

The data presented in this study are openly available in Figshare at https://doi.org/10.6084/m9.figshare.33036713.

## Supplementary Materials

The following supporting information can be downloaded at: https://www.mdpi.com/article/10.3390/healthcare14203524/s1, Material S1: Survey questionnaire.
