# Supplementary material for: ASAS Versus Modified New York Criteria for Ankylosing Spondylitis and Axial Spondyloarthritis: Attitudes and Utilization Among Saudi Rheumatologists
Source: Healthcare (Basel). 2026 Oct 20;14(20):3524. doi: 10.3390/healthcare14203524 (PMC13704382; doi:10.3390/healthcare14203524)
Supplement: Supplementary file 1 [file healthcare-14-03524-s001.zip › Supplementary_Questionnaire.pdf]

Supplementary Material S1. Survey Questionnaire

ASAS versus Modified New York Criteria for Ankylosing Spondylitis and Axial Spondyloarthritis: Attitudes and Utilization Among Saudi Rheumatologists

Dear Participant,

You are invited to participate in a research study about ankylosing spondylitis and axial spondyloarthritis among rheumatologists in Saudi Arabia. The target group are rheumatology consultants, specialists, and fellows who are now practicing in Saudi Arabia. The study is conducted by the Department of Internal Medicine, College of Medicine, at Imam Mohammad Ibn Saud Islamic University. This survey has been previously validated and utilized in other studies. Participation is voluntary, and you can withdraw from the study at any point without giving reasons. We appreciate your time.

Consent

1. As a rheumatology consultant/specialist/fellow practicing in Saudi Arabia, do you agree to participate in the study?

- ○ Yes
- ○ No

General Information and Demographics

2. Age

Please write it in numbers.

3. Nationality

- ○ Saudi
- ○ Non-Saudi

4. Gender

- ○ Male
- ○ Female

5. As a rheumatologist practicing in Saudi Arabia, are you:

- ○ A consultant rheumatologist
- ○ A rheumatology specialist/registrar
- ○ A fellow

6. As a rheumatology consultant/specialist/fellow practicing in Saudi Arabia, which of the following regions is your practice location?

- ○ Central region
- ○ Eastern region
- ○ Western region
- ○ Northern region
- ○ Southern region

7. If you are a rheumatology consultant/specialist, how many years have passed since you completed rheumatology fellowship training?

- ○ 5 or less
- ○ 6-10
- ○ 11-15
- ○ 16-20
- ○ 21-25
- ○ 26-30
- ○ >30

8. How would you personally identify yourself as?

- ○ Exclusively researcher, no patient care
- ○ Mostly researcher >75%; some patient care, teaching or administrative responsibilities
- ○ Mostly patient care >75%; some research, teaching, or administrative responsibilities
- ○ Exclusively patient care
- ○ Retired
- ○ Other

Modified New York (mNY) Criteria

Please look at the modified New York criteria. Clinical criteria: low back pain and stiffness improving with activity for more than three months; limited lumbar spine motion in forward and lateral bending; limitation of chest expansion relative to age- and sex-matched normal values. Radiological criteria: sacroiliitis grade ≥2 bilaterally, or grade 3 to 4 unilaterally. Diagnosis of AS is made if the patient fulfils at least one radiological and one clinical criterion.

9. Do you think they're useful in clinical practice?

- ○ Strongly agree
- ○ Agree
- ○ Neutral
- ○ Disagree
- ○ Strongly Disagree

10. Do you use them in clinical practice?

- ○ Always (>90%)
- ○ Usually (55-90%)
- ○ Half (45-55%)
- ○ Sometimes (10-45%)
- ○ Never, Rarely (<10%)

Assessment of SpondyloArthritis international Society (ASAS) Criteria

Please look at the ASAS criteria. In patients with ≥3 months of back pain and age at onset <45 years: sacroiliitis on imaging plus ≥1 SpA feature; or HLA-B27 plus ≥2 other SpA features. SpA features include inflammatory back pain, arthritis, enthesitis (heel), uveitis, dactylitis, psoriasis, Crohn's/colitis, good response to NSAIDs, family history of SpA, HLA-B27, and elevated CRP.

11. Do you think they're useful in clinical practice?

- ○ Strongly agree
- ○ Agree
- ○ Neutral
- ○ Disagree
- ○ Strongly Disagree

12. Do you use them in clinical practice?

- ○ Always (>90%)
- ○ Usually (55-90%)
- ○ Half (45-55%)
- ○ Sometimes (10-45%)
- ○ Never, Rarely (<10%)

Radiological Findings

13. How do you assess for sacroiliitis on X-rays?

- ○ I look at the X-rays myself and determine sacroiliitis (yes or no), without depending upon a read by a radiologist
- ○ I look at the X-rays myself and determine sacroiliitis (yes or no), but depend on a radiologist's final reading
- ○ I mainly depend on the radiologist's report

14. Do you order a sacroiliac joint MRI routinely for diagnostic purposes if sacroiliitis IS seen on X-Ray?

- ○ Yes
- ○ No

15. Do you order a sacroiliac joint CT scan routinely for diagnostic purposes if sacroiliitis IS seen on X-Ray?

- ○ Yes
- ○ No

16. Do you order a sacroiliac joint MRI routinely for diagnostic purposes if sacroiliitis IS NOT seen on X-Ray?

- ○ Yes
- ○ No

17. Do you order a sacroiliac joint CT scan routinely for diagnostic purposes if sacroiliitis IS NOT seen on X-Ray?

- ○ Yes
- ○ No

18. In the last one year, how many patients have you diagnosed and labeled as non-radiographic Axial SpA?

- ○ None
- ○ Less than 5 patients
- ○ 5 patients or more

Features for Diagnosing axSpA

19. How would you rate the specificity of the following SpA features for diagnosing axSpA?

For each feature, select one: Very Specific / Somewhat specific / Non-specific.

- Inflammatory back pain
- Inflammatory peripheral arthritis
- Enthesitis (heel)
- Uveitis
- Dactylitis
- Psoriasis
- Crohn’s / colitis
- Good response to NSAIDs
- Family history

Final Assessment

20. Overall, do you think the Assessment of SpondyloArthritis international Society (ASAS) classification criteria for Axial SpA are specific enough for use in clinical trials?

- ○ Strongly agree
- ○ Agree
- ○ Neutral
- ○ Disagree
- ○ Strongly Disagree

21. Do you think there is a need to modify current classification criteria (ASAS) for Axial Spondyloarthritis?

- ○ Strongly agree
- ○ Agree
- ○ Neutral
- ○ Disagree
- ○ Strongly Disagree

22. Do you think there is a need to modify current classification criteria (ASAS) for Ankylosing Spondylitis?

- ○ Strongly agree
- ○ Agree
- ○ Neutral
- ○ Disagree
- ○ Strongly Disagree

23. Would you treat non-radiographic axial spondylitis different than radiographic axial spondyloarthritis?

- ○ Yes
- ○ No

Thank you for participating in our survey. We deeply appreciate your time and valuable contribution.
